# Effects of unsupervised walking on walk performance and functional mobility in individuals with chronic stroke: a blind randomized clinical trial

**DOI:** 10.1590/1516-3180.2024.0190.R2.26022025

**Published:** 2025-08-29

**Authors:** Ronaldo Rodrigues Borges, André Pontes-Silva, Sara Andrade Rodrigues, Túlio Luiz Banja Fernandes, Claudio de Oliveira Assumpção, Almir Vieira Dibai-Filho, Cristiano Teixeira Mostarda, Augusto Ribeiro de Oliveira, Christian Emmanuel Torres Cabido

**Affiliations:** I Bachelor of Physical Education, Postgraduate Program in Physical Education, Department of Physical Education, Universidade Federal do Maranhão (UFMA), São Luís (MA), Brazil; Researcher, Research Group on Physical Exercise, Health and Human Performance (ExeF:SDH), Universidade Federal do Maranhão (UFMA), São Luís (MA), Brazil.; II Bachelor of Physical Education, PhD Student, Postgraduate Program in Physical Therapy, Department of Physical Therapy, Universidade Federal de São Carlos (UFSCAR), São Carlos (SP), Brazil.; III Physical Therapist, Professor, Institute of Biological and Health Sciences, Universidade Federal de Mato Grosso (UFMT), Barra do Garças (MT), Brazil.; IV Bachelor of Physical Education, Professor, Postgraduate Program in Physiotherapy and Functionality, Department of Physical Therapy, Universidade Federal do Ceará (UFC), Fortaleza (CE), Brazil.; V Bachelor of Physical Education, Professor, Postgraduate Program in Physical Education, Department of Sports Sciences, Institute of Health Sciences, Universidade Federal do Triângulo Mineiro (UFTM), Uberaba (MG), Brazil.; VI Physical Therapist, Professor, Postgraduate Program in Physical Education, Department of Physical Education, Universidade Federal do Maranhão (UFMA), São Luís (MA), Brazil; Professor, Postgraduate Program in Adult Health, Universidade Federal do Maranhão (UFMA), São Luís (MA), Brazil.; VII Bachelor of Physical Education, Professor, Postgraduate Program in Physical Education, Department of Physical Education, Universidade Federal do Maranhão (UFMA), São Luís (MA), Brazil.; VIII Bachelor of Physical Education, Postgraduate Program in Physical Education, Department of Physical Education, Universidade Federal do Maranhão (UFMA), São Luís (MA), Brazil; Researcher, Research Group on Physical Exercise, Health and Human Performance (ExeF:SDH), Universidade Federal do Maranhão (UFMA), São Luís (MA), Brazil.; IX Bachelor of Physical Education, Professor, Postgraduate Program in Physical Education, Department of Physical Education, Universidade Federal do Maranhão (UFMA), São Luís (MA), Brazil; Professor, Research Group on Physical Exercise, Health and Human Performance (ExeF: SDH), Universidade Federal do Maranhão (UFMA), São Luís (MA), Brazil; Professor, Postgraduate Program in Adult Health, Universidade Federal do Maranhão (UFMA), São Luís (MA), Brazil.

**Keywords:** Stroke rehabilitation, Exercise, Public Health, Quality of life, Aerobic Exercise, Physical Activity, Rehabilitation, Walking Speed

## Abstract

**BACKGROUND:**

What are the effects of walking training on the ground in an unsupervised manner and with different weekly durations after chronic stroke?

**OBJECTIVE:**

To compare the effects of unsupervised walking for 150 and 300 minutes per week on walking performance, speed, and functional mobility in individuals with chronic stroke.

**DESIGN AND SETTING:**

Randomized clinical trial was conducted at Rede Sarah Rehabilitation Hospital (São Luís, Brazil).

**METHODS:**

Individuals included (n = 40) were assessed using the 6-minute walk test (6MWT), functional mobility using the Timed Up and Go (TUG) test, and the Five Times Sit to Stand Test (FTSST). They were assigned to the two experimental groups and instructed to walk 150 (G150) or 300 minutes per week (G300) and to perform unsupervised gait training for the next eight weeks.

**RESULTS:**

No significant differences were observed between the group factors and no significant interaction was found for the group × time interaction, indicating that G150 and G300 changed similarly. The comfortable walking speed increased for both G150 and G300, resulting in a large effect size. Performance on the TUG and 6MWT also improved, but the effect size was small. For maximum walking speed, despite the improvement in performance in G150 and the G300, effect size was medium for both groups. The same was true for the FTSST.

**CONCLUSION:**

Unsupervised walking was effective in improving gait performance and functional mobility in individuals with chronic stroke regardless of the recommended weekly duration (150 or 300 minutes).

**CLINICAL TRIAL REGISTRATION:**

RBR-5g4g9bq (https://ensaiosclinicos.gov.br/rg/RBR-5g4g9bq).

## INTRODUCTION

 Studies have evaluated the contribution of gait training to safe and independent walking in individuals with chronic stroke, showing improvements in comfortable and maximal walking speed (determined from the 10-meter walk test),^1-5^ walking endurance as measured by the distance covered in the 6-minute walk test (6MWT),^1-6^ and functional mobility as measured by the time spent performing the Timed Up and Go (TUG) test.^7,8^ Less commonly, the task of standing up from a seated position has also been used to measure functional mobility after exercise programs, with assessment using the Five Times Sit to Stand Test (FTSST).^9^

 Regarding exercise volume, which is also prescribed as a weekly duration, guidelines for adults after stroke suggest positive outcomes, even with protocols that vary in weekly duration. For example, the American Stroke Association, one of the main guidelines for stroke, recommends a weekly duration of 150 to 300 minutes of light-to-moderate intensity exercise.^10,11^ Although there is evidence that exercise volume positively correlates with walking performance after stroke,^7^ studies using walking as an intervention used volumes close to or equal to 150 minutes per week; a minimum amount is also recommended to reduce sedentary lifestyle and its complications.^5,12,13^

 However, although the weekly duration of 150 minutes has been shown to be effective in these studies, the training programs were conducted with specific walking equipment (treadmills) and/or with close professional supervision, factors that make it difficult for people to implement and maintain the exercises of individuals who have already completed rehabilitation programs.^11^ Unsupervised walking without the use of equipment is expected to allow for greater patient engagement, but with the disadvantage that the exercise load performed may be less than planned because of the lack of direct monitoring.^7,10^

 We hypothesized that unsupervised walking would be as effective as supervised walking in individuals with chronic stroke. Therefore, we compared the effects of unsupervised walking for 150 or 300 minutes per week on walking performance (6MWT),^6^ speed, and functional mobility (TUG^14,15^ and FTSST^9^) of individuals with chronic stroke. 

## METHODS

### Trial design and ethical aspects

 Randomized clinical trial was according to CONSORT.^16^ Participants were evaluated at three time points: clinical enrolment, pre-intervention, and post-intervention. This study was approved by the Brazilian Registry of Clinical Trials (report number: RBR-5g4g9bq) and the Research Ethics Committee (report number: 3739370). Data collection for the current study was performed during routine care at the Rede Sarah de Hospitais, São Luís, Brazil, from May 20, 2019, to December 31, 2020. 

 Individuals were evaluated at regular follow-up visits and in the hospital on three different days: on admission, before the intervention (pre), and at the end of the intervention (post). On admission, after the assessment of cardiovascular risk factors under the guidance of a clinical physician, an exercise specialist (the investigator of this study) performed tests to assess comfortable and maximal walking speed using the 10-meter sprint test,^6^ walking endurance using the 6MWT,^6^ and functional mobility using the TUG test^14,15^ and FTSST.^9^

 After the initial assessments, individuals were advised to return home, maintain their usual routine, and return to the hospital after eight weeks (review care). This eight-week interval was used to carry out the randomization of the sample, to allow for the availability of places for team care, and for the logistical organization of the use of institutional space. No interventions were administered during this period. 

 After the first eight weeks (period of no intervention since admission), individuals returned for review, re-evaluation, and instruction (pre). On this day, they were instructed to perform unsupervised gait training for the next eight weeks according to the instructions provided, knowing only the gait protocol they were to perform. The unsupervised walking training period was identical for both experimental groups. At the end of these eight weeks of unsupervised walking training, individuals returned for a new review for reassessment and final instruction (post). 

### Participants

 Individuals included were in the normal course of care at the hospital; that is, they had already undergone some form of follow-up with the rehabilitation team at some point after stroke. Inclusion criteria for the sample were as follows: diagnosis of a stroke that occurred at least 6 months ago, supported by a medical report; and comfortable walking speed (CWS) of less than 1.0 m/s, i.e., limited (0.4 m/s to 0.8 m/s) and unrestricted (0.8 m/s to 1.0 m/s) community ambulators.^10,11^

 Speed was measured on the day of admission by walking time in the 10-meter test;^6^ having participated in a motor rehabilitation program at any time after the stroke; and not having participated in any physical training or rehabilitation programs during the study period or in the previous six months. The following conditions were considered as sample exclusion criteria: any clinical condition that made it impossible to perform examinations and assessments; inability to walk at least 10 meters without assistance;^6^ and cognitive deficits that made it difficult to understand simple verbal commands, as identified by previous medical records using the Mini-Mental State Examination with a score below 23 points.^10,11^

 The individuals who were unable to complete at least 80% of their planned weekly volumes during the training period were excluded. This methodological criterion was adopted to increase the possibility of expected changes in performance depending on the totality of the oriented durations. Furthermore, individuals excluded by the exclusion criteria would participate without distinction in all care throughout the follow-up period so that their rehabilitation program would be identical to that of the others.^10,11^

### Randomization

 Stratified randomization was performed by a third party blinded to the research content and was used to reduce group heterogeneity due to differences in walking speed, despite the fact that all individuals met the inclusion criteria.^17^ For this process, individuals were listed and classified according to their CWS (measured by the 10-meter test on the day of admission) as limited community ambulators (speed between 0.4 m/s and 0.8 m/s) or unlimited (speed greater than 0.8 m/s). 

**Figure 1:**
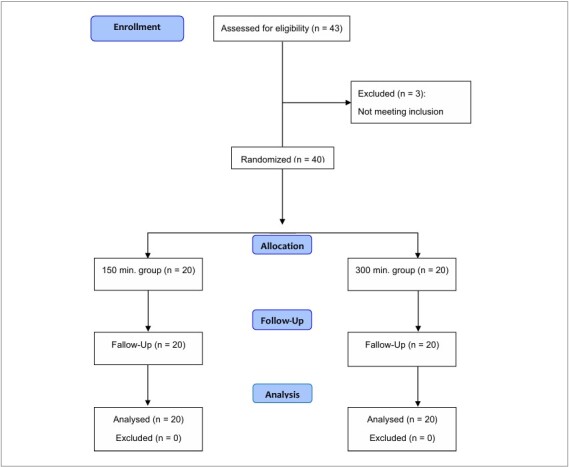
Flowchart.

 Half of the individuals in each classification were then randomly assigned to two experimental groups and instructed to walk for 150 minutes (G150) or 300 minutes (G300) per week. Thus, with stratified randomization, both groups had similar proportions of limited and unlimited community ambulation (Figure 1). 

### Interventions

 The unsupervised walking training lasted for eight weeks and was conducted in the community. Individuals were instructed on the correct use of medications, best time and place to walk (avoiding uneven terrain, excessive sun exposure, and high temperatures), and the need for adequate hydration and nutrition. Safety and fall prevention should be prioritized using mobility devices (if used). 

 Each group was instructed to perform one of the two proposed weekly durations of unsupervised walking (150 or 300 minutes). The existence of a second protocol was not disclosed; that is, each group knew only the exercise program they were to perform. The G150 group was asked to walk for 30 minutes per day (five days per week), a methodological criterion adopted to equalize the frequency of training between the groups, thus allowing a regular distribution of the prescribed durations throughout the week. They were instructed to maintain the highest sustainable walking speed as exercise intensity, an effective parameter to promote functional recovery in individuals with chronic stroke.^18^ The G300 group was instructed to walk 60 minutes per day (five days per week). 

 To determine the effort exerted, individuals were asked to record the Borg Rating of Perceived Exertion (RPE) scale during each training session in a diary prepared and provided by the team on the second day of the assessment (pre).^19,20^ The RPE scale^19,20^ was presented to them in detail on that day, with the score reinforced based on the verbal anchors of the instrument, emphasizing and exemplifying the initial anchor (no effort) and final anchor (maximum effort already made). The use of diaries is common for monitoring training performed outside of rehabilitation centers.^21^ This diary included all possible exercise days during the eight-week period and was accompanied by a RPE.^19,20^ Individuals were asked to complete it by answering three items: "Put an ‘X’ on the day you exercised"; "How many minutes did you walk?"; "Perceived exertion (rate from 6 to 20)." The intensities were considered light (RPE < 11), moderate (RPE 11–14), or high (RPE > 14). 

 Another methodological criterion adopted in accordance with the guidelines was the possibility of delivering training continuously or at regular intervals. Since the individuals had not participated in training programs during the previous six months, they may have had low exercise tolerance, which would reduce their commitment. Therefore, the suggested durations could be performed continuously in each session or with minimal rest intervals (if necessary), as long as a minimum duration of 10 minutes was maintained in each effort period, thus maintaining the aerobic nature of the exercise.^22^ Walking intensity and RPE^19,20^ recording guidelines were the same as those recommended for the G150.^10,11^

### Outcomes – blinded

 The evaluations were performed by a third party who was unaware of the research content. Individuals could count on the presence of one or more companions/family members to perform the tests, and those who use mobility aids (canes/walkers) and/or assistive devices (orthotics and insoles) should maintain their use during assessments for safety and to reduce the risk of falling. All the tests were explained in advance by the researcher. The primary variables assessed were walking performance based on comfortable speed and walking endurance (distance walked in the 6MWT)^6^ and functional mobility (time taken to complete the TUG test).^14,15^

 Secondary variables included performance on a functional test of sitting and standing five times, and maximum walking speed. For tests that required precision from the examiner through time tracking (10 m sprint test,^6^ TUG,^14,15^ and FTSST^9^), the reliability of the intra-rater measurements was determined, which guaranteed an excellent intraclass correlation coefficient (ICC) for all variables (10 m sprint test:^6^ ICC = 0.983; TUG:^14,15^ ICC = 0.986; FTSST:^9^ ICC = 0.953). 

 Gait performance was assessed using the 10-m sprint test, which consisted of measuring the time taken to walk 10 m in a straight line, starting from the starting position in a twometer area and ending in a similar area.^6^ This test is valid and reliable for measuring the CWS and maximum walking speed of individuals with chronic stroke.^7^ Individuals performed three repetitions at the walking speed they considered comfortable (self-regulated) and three repetitions at the highest possible speed (maximum), with a one-minute recovery interval between each repetition. Prerepetition was performed to resolve any uncertainties. 

 Walking endurance was measured using the 6MWT.^6^ The test consists of measuring the longest distance walked over a six-minute period using a 30-meter bounded space.^6^ Only one repetition was performed, as this is a more physically demanding test. This test is also used to indirectly assess the aerobic capacity in individuals with various clinical conditions, including the elderly, healthy individuals, and individuals with stroke. This is reliable for measuring walking endurance in people with chronic stroke.^7^ Before and after the 6MWT^6^ vital signs were measured using heart rate and blood pressure, and intensity was measured using the RPE.^19,20^

 Functional mobility was measured using the TUG test.^14,15^ The test measures the time taken to rise from a sitting position in a chair to an upright position, walk three meters, return, and sit down again. This is a valid and reliable test for measuring functional mobility in patients with chronic stroke.^7^

 The ability to move from a sitting to a standing position was considered a secondary variable in this study. This was measured using the FTSST,^9^ which requires the participants to stand and sit down five times in the shortest possible time, starting from a sitting position in a chair. The FTSST^9^ is a valid and reliable measure of functional mobility based on the assessment of transfer from sitting to standing in individuals with stroke.^8^ Three repetitions were performed with a one-minute recovery interval, and the mean time was recorded. Each participant performed one repetition to resolve any questions. 

### Sample size

 The sample consisted of 40 adults (aged 18 years or older) with hemorrhagic and/or ischemic stroke of both sexes (31 men) who used or did not use devices to facilitate gait (orthoses and inserts) and mobility (cane and walker). The sample calculation was performed using GPower 3.1 software (Heinrich Heine University, Düsseldorf, Germany), considering repeated measures comparisons between factors (time and group) with a minimum effect size of 40%, observed power of 90% (1- β = 0.90), and index significance level of 5% (α of 0.05), resulting in a sample size of 36 individuals.^7^ However, to account for potential sample loss throughout the process, 43 individuals were included, of which three were excluded based on the imposed criteria, resulting in a final sample of 40 individuals. 

### Statistical methods

 We performed an intention-to-treat analysis^23^ using SPSS version 24.0. The Shapiro–Wilk test was used to test the normality assumption of the variables. The t-test, chi-square test, and/or Fisher’s exact test were used to compare descriptive statistics depending on the level of the variables. A generalized estimating equation model was used to compare the quantitative variables in each experimental group (G150 and G300) in the pre- and post-intervention situations.^24^ Time and group were considered as independent fixed effect factors and Bonferroni post hoc test was used when necessary (significance level used was P < 0.05). 

 Effects of independent fixed factors and parameter estimates from generalized estimating equations were described using Wald comparison values (W-value: values > 1 indicate a significantly estimated probability), P value, and difference of means (B value) with a 95% confidence interval (95% CI).^24^ The effect size was also determined using Cohen’ d for paired groups.^25^

 For tests that required the precision of the examiner by timing (10 m sprint test,^6^ TUG,^14,15^ and FTSST^9^), the intra-rater reliability of the measurements was determined using the Wilcoxon test followed by Spearman’s correlation and determination of the ICC. The two-way absolute agreement model was used for this calculation, with the mean of the three trials used as the criterion for time determination.^26,27^ Reliability was interpreted as low (< 0.40), moderate (0.40 to 0.75), substantial (0.75 to 0.90), or excellent (> 0.90).^25^

## RESULTS

 The sample characteristics and exercise session records are in Tables 1 and 2. In the comparison carried out using the generalized estimating equation model (Table 3), it was determined that only the time factor was influenced by the proposed intervention, significantly modifying all dependent variables evaluated before and after the prescription of unsupervised walking. No significant differences were observed between the group factors and no significant interaction was found for the group × time interaction, indicating that G150 and G300 changed similarly. 

**Table 1:** Sample characterization. Values are described as mean (± SD) or % (absolute number)

**Variables**		**150 min. group (n = 20)**	**300 min. group (n = 20)**
**Age (years)**		52 (± 14)	58 (± 10)
**Sex (male)**		80% (16)	75% (15)
**Time since stroke (months)**		44 (± 34)	39 (± 33)
**Walking speed (m/s)**		0.69 (± 0.22)	0.69 (± 0.25)
**Mobility aid**		70% (14)	65% (13)
**Stroke type**			
	Ischemic	75% (15)	85% (17)
	Hemorrhagic	25% (5)	15% (3)
**Affected brain region**			
	Brain	75% (15)	80% (16)
	Cerebellum	20% (4)	5% (1)
	Stem	5% (1)	15% (3)
**Spasticity ^a^**			
	Degree 0	25% (5)	30% (6)
	Degree 1	20% (4)	15% (3)
	Degree 1+	35% (7)	40% (8)
	Degree 2	20% (4)	15% (3)
**Hemiparetic side**			
	Right	40% (8)	70% (14)
	Left	60% (12)	30% (6)
**Risk factors**			
	Systemic Arterial Hypertension	85% (17)	80% (16)
	Dyslipidemias	60% (12)	85% (17)
	Obesity	30% (6)	20% (4)
	Smoking	25% (5)	15% (3)
	Diabetes	20% (4)	20% (4)
**Medications in use**			
	Antihypertensives	80% (16)	80% (16)
	Antispastic	50% (10)	50% (10)
	Hypoglycemic	15% (3)	20% (4)
	ypocholesterolemia	10% (2)	70% (14)

^a^ degree of spasticity in the lower limbs, as measured using the modified Ashworth scale. None of the comparisons showed significant differences (P ≥ 0.05).

**Table 2:** Records made during exercise sessions

**Variables**		**150 min. group (n = 20)**	**300 min. group (n = 20)**
**Borg Rating of Perceived Exertion (RPE) scale**			
	Light (< 11)	10% (2)	0
	Moderate (11–14)	85% (17)	80% (16)
	High (> 14)	5% (1)	20% (4)
**Realized volume in relation to proposed**			
	Full (100%)	90% (18)	90% (18)
	Partial 1 (80%–99%)	10% (2)	10% (2)
	Partial 2 (< 80%)	0	0

RPE = Borg Rating of Perceived Exertion. None of the comparisons showed significant differences (P ≥ 0.05).

**Table 3:** Effects of independent factors and parameter estimates

**Variables**	**Factors**	**W value^a^**	**P value^b^**	**B value^c^**	**95% CI**
**CWS (m/s)**	Group	0.091	0.763	0.043	-0.111, 0.198
	Time	33.677	< 0.001*	-0.258	-0.397, -0.120
	Group × Time	0.269	0.604	0.051	-0.242, 0.141
**MWS (m/s)**	Group	0.034	0.854	-0.024	-0.106, 0.388
	Time	16.595	< 0.001*	-0.267	-0.424, -0.110
	Group × Time	0.490	0.484	0.078	-0.141, 0.298
**TUG (s)**	Group	0.061	0.804	-0.025	-0.276, 0.226
	Time	23.869	< 0.001*	0.176	0.114, 0.238
	Group × Time	0.408	0.235	0.113	-0.074, 0.300
**6MWT (m)**	Group	0.094	0.759	-0.022	-0.191, 0.148
	Time	73.638	< 0.001*	-0.180	-0.254, -0.106
	Group × Time	0.104	0.747	-0.014	-0.099, 0.071
**FTSST (s)**	Group	0.414	0.520	-0.072	-0.252, 0.108
	Time	96.300	< 0.001*	0.321	0.218, 0.420
	Group × Time	0.014	0.905	0.008	-0.122, 0.138

CWS = Comfortable walking speed; MWS = Maximum walking speed; TUG = Timed up and go test; 6MWT = 6-min walk test; FTSST = Five times sit to stand test.

^a^ Comparison of means estimated by generalized estimating equations (Wald value).

^b^ Significant difference (P < 0.05).

^c^ Difference from estimated means with confidence interval (95% CI).

* P < 0.001.

^b,c^ = Generalized estimating equations for longitudinal data analysis – time and group were considered independent fixed effect factors, and Bonferroni post hoc was used when necessary (Ballinger, 2024).^24^

 Estimated mean for CWS increased for the G150 (from 0.69 m/s ± 0.22 m/s to 0.94 m/s ± 0.33 m/s) and for the G300 (from 0.69 m/s ± 0.25 m/s to 0.90 m/s ± 0.32 m/s), resulting in a large effect size for both (G150, Cohen d = 0.92; G300, 1.10). Performance on the TUG and 6MWT also improved, but the effect size was small. On the TUG, the time reduced for the G150 group from 12.65 s ± 8.38 s to 9.47 s ± 5.16 s (Cohen d = 0.47) and the G300 group from 11.58 s ± 7.12 s to 9.71 s ± 5.82 s (Cohen d = 0.29). For the 6MWT, the distance increased for the G150 group from 333.00 m ± 140.73 m to 404.30 m ± 163.17 m (Cohen d = 0.47) and the G300 group from 345.20 m ± 184.04 m to 413.25 m ± 152.43 m. 

 Regarding maximum walking speed, despite the improvement in performance for G150 (from 1.00 m/s ± 0.46 m/s to 1.27 m/s ± 0.57 m/s; Cohen d = 0.53) and G300 (from 0.98 m/s ± 0.40 m/s to 1.28 m/s ± 0.58 m/s; Cohen d = 0.61), the effect size was medium for both groups. Likewise, for the FTSST (G150: from 11.25 s ± 5.3 s to 8.10 s ± 2.35 s, Cohen d = 0.79; G300: 8.70 s ± 4.38 s, Cohen d = 0.55). 

## DISCUSSION

 Among the variables evaluated, CWS, which is an important parameter to characterize independent walking performance^28,29^ and is also used to classify poststroke walking ability,^30^ increased similarly in both groups (G150: 0.94 m/s; G300: 0.90 m/s), resulting in large effect size (Cohen d > 0.8).^25^ Thus, the results of the present study demonstrated that even in an unsupervised manner, walking training of 150 or 300 minutes per week significantly increased the CWS of individuals to the point of becoming community walkers^31^ limited to unlimited, thus contributing positively to their level of independence.^32^

 Charalambous et al. suggested that the clinical significance of a CWS would only be achieved after 12 weeks of training.^33^ However, in addition to using a weekly duration of 90 minutes, which is less than recommended for health benefits,^10,11^ they walked on a treadmill combined with electrical stimulation of the ankle muscles. In the present study, clinical relevance was achieved with a large effect size for the same variable and in a shorter time period (eight weeks), with walking training performed on the ground in an unsupervised manner, and with a weekly duration of physical activity sufficient to provide health benefits. 

 Performance on the TUG,^14,15^ an important test to assess functional mobility after stroke, improved in both groups after the intervention (G150: 9.7 s, effect size = 0.47; G300: 9.71 s, effect size = 0.29), but with little clinical relevance as the effect size was small.^25^ Similarly, performance on the 6MWT,^6^ although improved, was of little clinical relevance, with a small effect size (G150: 404.30 m, effect size = 0.47; G300: 413.25 m, effect size = 0.41). 

 Among the secondary variables, maximum walking speed, which measures the ability to walk quickly over short distances, showed a significant increase (P < 0.001) with a medium effect size in both groups. Performance on the FTSST^9^ also improved significantly (P < 0.001), with a reduction in test performance time and average effect size for both groups, similar to that found for maximum walking speed. To date, no values of clinical importance have been found for these two variables, which would allow for comparisons with other training programs. 

 Maintaining a regular walking routine for eight weeks, even if unsupervised, may have provided a training overload capable of stimulating neuroplasticity, increasing the recruitment of motor modules, and improving muscle synergy.^34^ Consequently, greater muscular synergy or synchronization of motor units may have contributed to increases in explosive strength, balance, and skills required in the tests performed and in everyday tasks.^32,34^

 Another explanation for the benefits of walking could be related to the reduction in the co-activation time of the lower limb muscles, which would lead to better intramuscular coordination, as has already been shown in training with the same exercise.^35^ Among these possible neural adaptations that occurred, the synchronization of motor units was perhaps the most prevalent with running training. This could also explain the greater performance gains in tests that mainly demand explosive strength (maximum walking speed and FTSST^9^) compared to the TUG^14,15^ and 6MWT,^6^ which predominantly demand other abilities, such as balance and resistance, respectively. Regarding the proposed protocols, moderate effort was most frequently reported by individuals in unsupervised training sessions, which is compatible with the intensity recommended in the literature.^10,11^

 Furthermore, the individuals already had a high performance of functional mobility and walking endurance at the time of enrolment, with values higher than those of other studies for the TUG^14,15^ and 6MWT,^6^ respectively.^18^ A possible explanation for this better baseline performance in the present study could be the fact that individuals had already participated in a post-stroke rehabilitation program, although it was not possible to quantify the previously spent rehabilitation time. This would also explain the smaller effect sizes for TUG^14,15^ and 6MWT,^6^ as the baseline performance was already high and little could change with the intervention performed. 

 Studies that used walking as an exercise in the training program and found improvements in gait performance and functional mobility used volumes close to or equal to 150 minutes per week,^18^ but they used treadmills and/or close professional supervision. This distinguishes this study, which also found interesting results when walking training was performed on the ground and unsupervised. 

 The proposed two-weekly training duration improved all variables evaluated, including those of great clinical importance for CWS. Therefore, professionals involved in the rehabilitation of stroke patients may find that 150 minutes of weekly walking in a home/community setting is sufficient because it requires little daily time and keeps individuals physically active as recommended.^10,11^

## CONCLUSION

 Unsupervised walking was effective in improving the gait performance and functional mobility of individuals with chronic stroke regardless of the recommended weekly duration (150 or 300 minutes). Therefore, walking for 150 minutes a week may be sufficient to improve gait and functional mobility in individuals with chronic stroke who have already participated in rehabilitation programs. 
